# Cronkhite-Canada syndrome associated with rib fractures: a case report

**DOI:** 10.1186/1471-230X-10-121

**Published:** 2010-10-18

**Authors:** Bosi Yuan, Xinxin Jin, Renmin Zhu, Xiaohua Zhang, Jiong Liu, Haijun Wan, Heng Lu, Yunzhu Shen, Fangyu Wang

**Affiliations:** 1 Department of Gastroenterology, Jinling Hospital, Jiangsu province, China

## Abstract

**Background:**

Cronkhite-Canada syndrome (CCS) is a rare multiple gastrointestinal polyposis. Up till now, many complications of CCS have been reported in the literature, but rib fracture is not included.

**Case Presentation:**

We report a case of a 58-year-old man who was admitted to our hospital with a 6-month history of frequent diarrhea, intermittent hematochezia and a weight loss of 13 kg. On admission, physical examination revealed alopecia of the scalp, hyperpigmentation of the hands and soles, and dystrophy of the fingernails. Laboratory data revealed hypocalcaemia and hypoproteinemia. Esophagogastroduodenoscopy, video capsule endoscopy and colonoscopy revealed various sizes of generalized gastrointestinal polyps. Histological examination of the biopsy specimens obtained from the stomach and the colon showed adenomatous polyp and inflammatory polyp respectively. Thus, a diagnosis of CCS was made. After treatment with corticosteroids for 24 days and nutritional support for two months, his clinical condition improved. Two months later, he was admitted to our hospital for the second time with frequent diarrhea and weight loss. The chest radiography revealed fractures of the left sixth and seventh ribs. Examinations, including emission computed tomography, bone densitometry test, and other serum parameters, were performed, but could not identify the definite etiology of the rib fractures. One month later, the patient suffered from aggravating multiple rib fractures due to the ineffective treatment, persistent hypocalcaemia and malnutrition.

**Conclusions:**

This is the first case of a CCS patient with multiple rib fractures. Although the association between CCS and multiple rib fractures in this case remains uncertain, we presume that persistent hypocalcaemia and malnutrition contribute to this situation, or at least aggravate this rare complication. Besides, since prolonged corticosteroid therapy will result in an increased risk of osteoporotic fracture, CCS patients who accept corticosteroid therapy could be potential victims of rib fracture.

## Background

Cronkhite-Canada syndrome (CCS) is a rare acquired polyposis syndrome characterized by multiple gastrointestinal polyps with alopecia, nail dystrophy, and hyperpigmentation. The syndrome was first reported in 1955 by Cronkhite and Canada [1]. Since then, more than 400 cases of Cronkhite-Canada syndrome had been reported worldwide, with 75% of them coming from Japan [2]. The mean age of onset is reported to be 59 years, and the male to female ratio is 3:2 [3]. So far, no definite etiology of CCS has been determined, though mental stress or physical fatigue is thought to be involved [4], and no strong evidence to suggest a familial predisposition. Moreover, various kinds of concomitant diseases of CCS have been reported. Here we describe a case of CCS in a patient with multiple rib fractures.

## Case Presentation

A 58-year-old man was admitted to our hospital with a 6-month history of frequent watery diarrhea (10-20 times per day), intermittent hematochezia, and a weight loss of 13 kg. Two months after onset of symptoms, he noticed pigmentation in the palms and hair loss. He had a negative family history of gastrointestinal disease and congenital disease. On physical examination, the patient was found to have marked alopecia, brownish macular pigmentation over the palms and soles, and onychodystrophy of the fingernails. The remainder of the physical examination was unremarkable.

**Figure 1:**
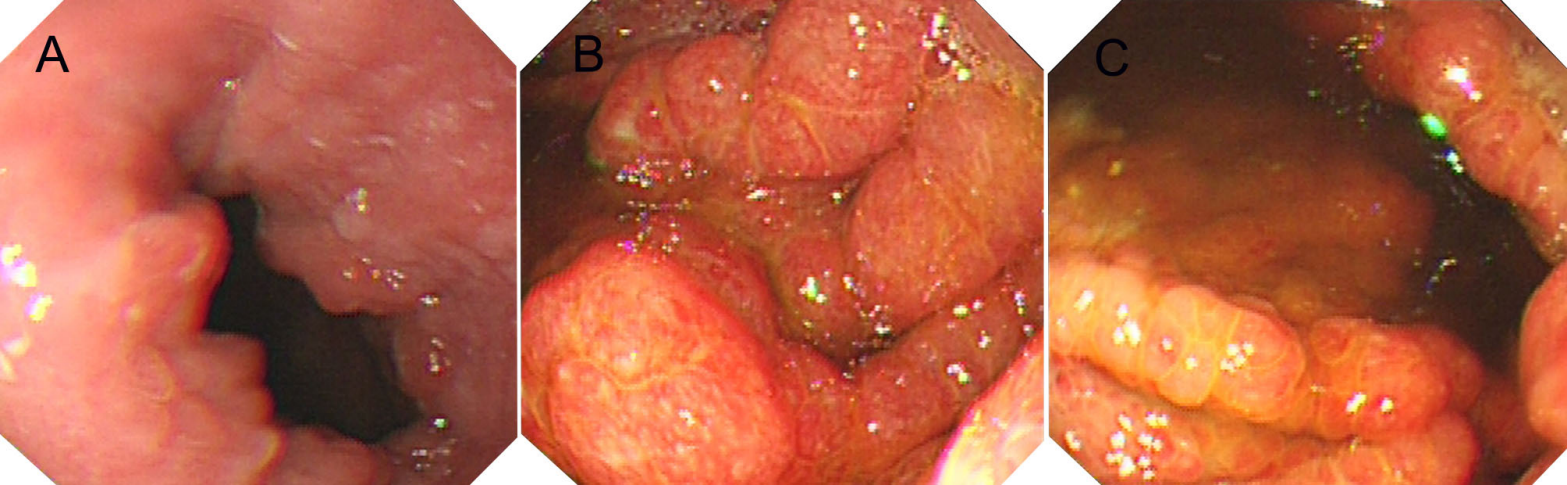
**Endoscopic views**. Esophagogastroduodenoscopy showed diffuse polypoid lesions extending from cardia to the first part of the duodenum. A, Cardia; B, Stomach; C, Duodenum.

**Figure 2:**
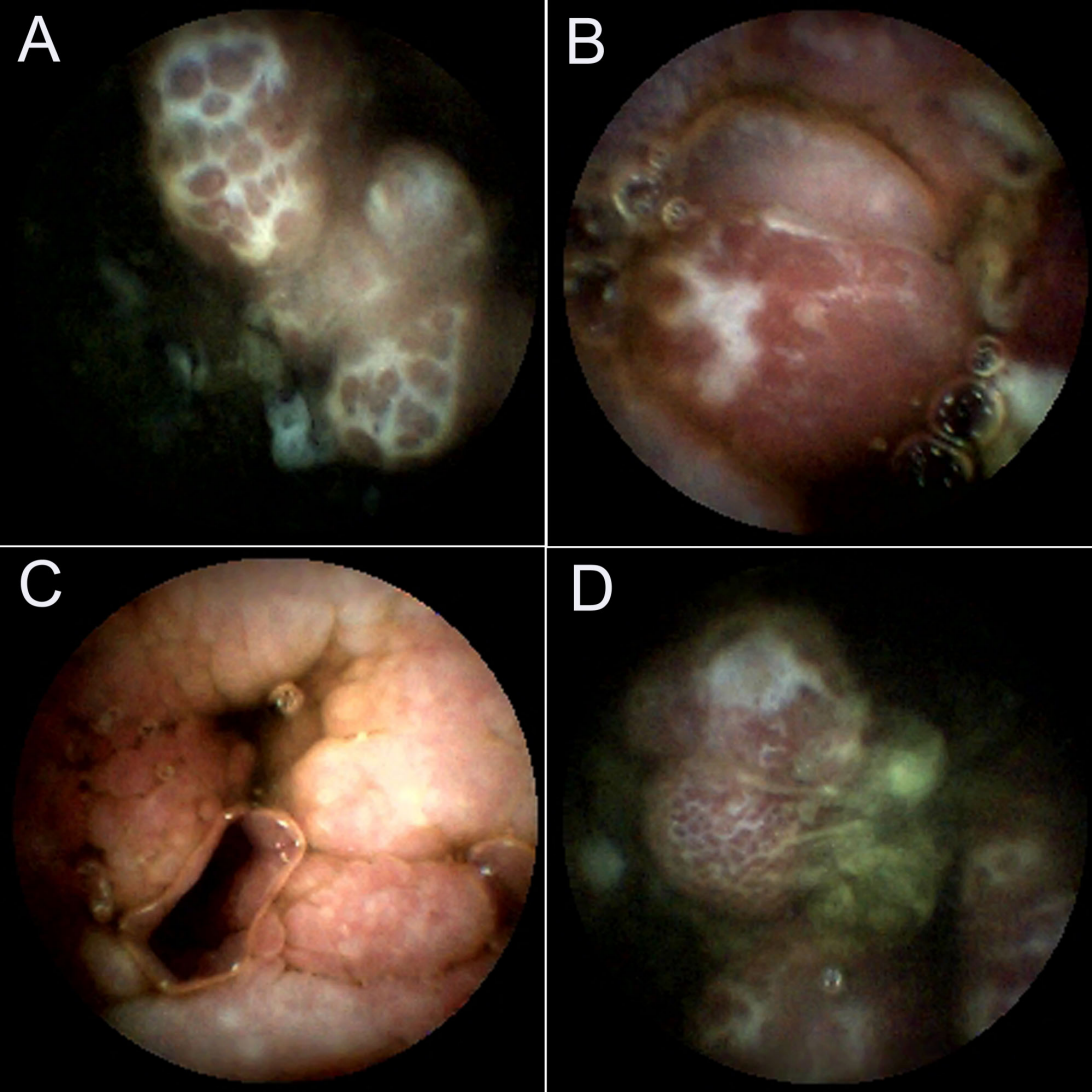
**Endoscopic views**. Video Capsule Endoscopy showed multiple herpes-like and strawberry-like polyps studded in most of the jejunum and ileum. A and B, Jejunum; C and D, Ileum.

**Figure 3:**
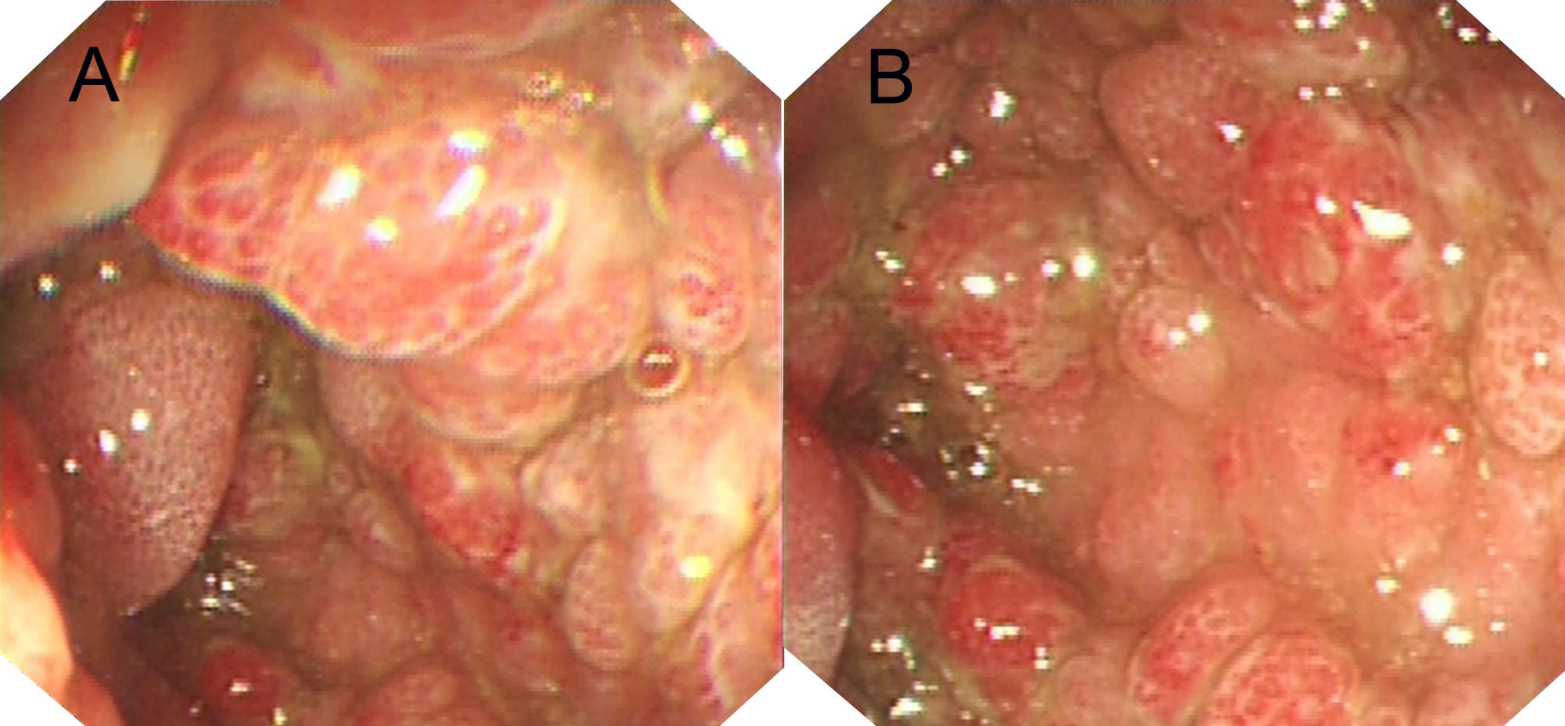
**Endoscopic views**. Colonoscopy identified numerous, hyperemic, sessile and pedunculated polyps in the colorectum. A, Descending Colon; B, Transverse Colon.

Initial laboratory data showed that his albumin level was 31.2 g/L (normal range 35-55 g/L), serum potassium 3.1 mmol/L (normal range 3.5-5.5 mmol/L) and serum calcium 1.7 mmol/L (normal range 2.1-2.6 mmol/L). Other blood parameters, including thyroid hormones, parathyroid hormone and immunoglobulins, were within the normal range. The chest radiograph was negative. Esophagogastroduodenoscopy, video capsule endoscopy and colonoscopy were performed for further evaluation of the gastrointestinal tract and they identified various sizes of generalized gastrointestinal polyps (Figures 1, 2 and 3). Histological examination of the biopsy specimens obtained from the stomach and the colon showed adenomatous polyp and inflammatory polyp respectively. Thus, a diagnosis of CCS was made. We started corticosteroid therapy for him with oral prednisone (40 mg per day for 2 weeks and then reduced the dosage to 30 mg per day and lasted it for 10 days), but then we discontinued it because the clinical situation of the patient became better. At the same time, the patient was treated with nutritional supplementation by parenteral and enteral nutrition. His situation improved gradually after two months of treatment. The frequency of diarrhea decreased to 2 times per day, the weight increased by 5 kg, and the hair and fingernails regrew, but the levels of serum calcium (1.9 mmol/L) and albumin (30.6 g/L) were still lower than the normal range. He returned home for home nutritional support by enteral nutrition.

**Figure 4:**
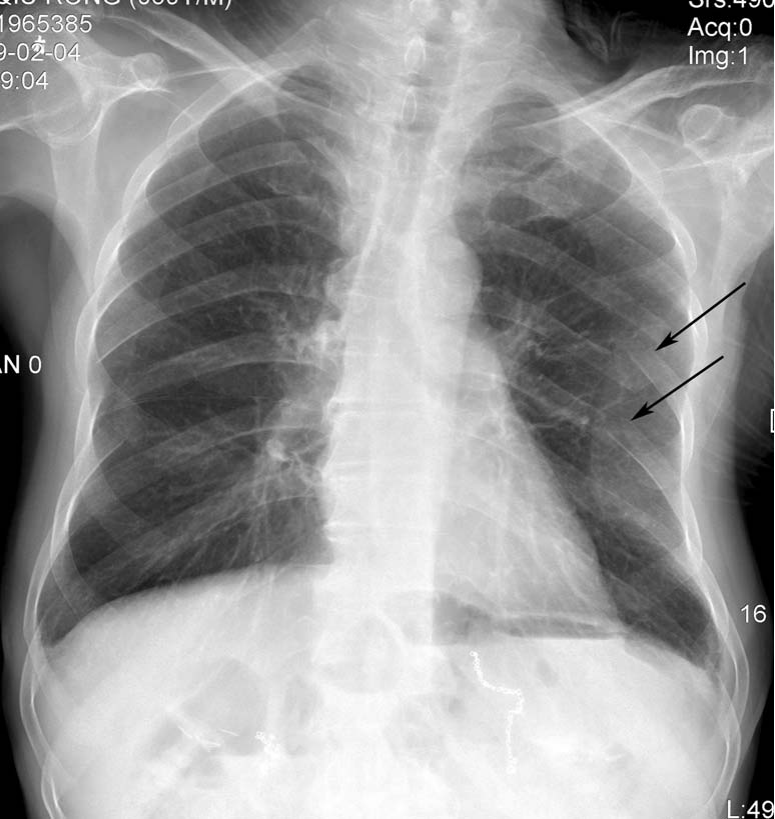
**X-ray image**. A chest radiograph showed fractures of the left sixth and seventh ribs (arrow).

Two months later, he was admitted to our hospital for the second time with frequent diarrhea (7-8 times per day) and a weight loss of 7 kg. Laboratory data showed that his serum albumin level was 28.2 g/L and serum calcium 1.7 mmol/L. His chest radiograph showed fractures of the left sixth and seventh ribs (Figure 4). Since the patient had not suffered from any load or trauma in the chest, this concomitant complication initially led us to presume that there was a possibility of rib metastasis of a malignant tumor. Emission Computed Tomography (ECT) was performed and it showed no increased tracer uptake in the skeletal system. Bone densitometry tests on vertebrae lumbales and caput femoris were normal. Further examination for checking bone metastasis was not performed because of the patient's financial situation, and his examinations and clinical features indicated no definite malignant tumor. Since his nutritional status was poor and he had no complain of pain in chest, orthopedic surgeons and chest surgeons advised us to supply calcium and nutrition for him and to restrict his chest wall movement. After one-month treatment, which was similar to our previous treatment except for the corticosteroid therapy, his clinical condition markedly improved again. His serum albumin level increased to 35.2 g/L and calcium to 1.9 mmol/L. However, the rib fractures persisted.

**Figure 5:**
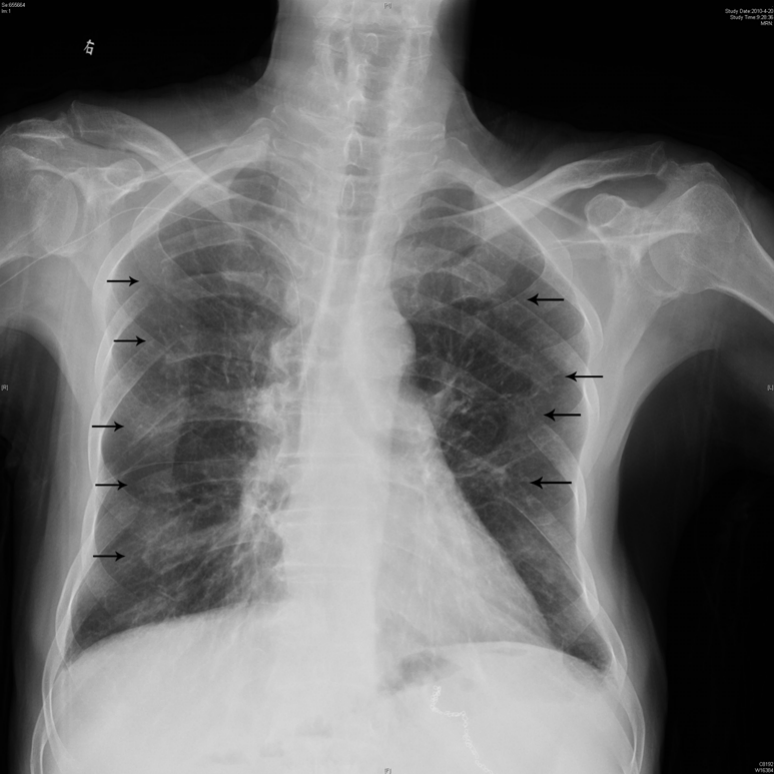
**X-ray image**. A chest radiograph showed multiple fractures of the left fourth to seventh ribs (arrow) and right fifth to ninth ribs (arrow).

After one-month of home nutritional support, he was admitted to our hospital for the third time to improve his nutritional status. His chest radiograph revealed aggravating multiple rib fractures (Figure 5). Since he did not complain of chest pain and respiratory distress when walking or resting, we consulted the orthopedic surgeons and chest surgeons again and received the same treatment recommendations. After one-month combination therapy, based on nutritional support, his weight increased by 4.5 kg, but hypocalcaemia (calcium level, 1.9 mmol/L), hypoalbuminemia (albumin level, 30.9 g/L) and multiple rib fractures still persisted.

## Discussion

CCS has been reported over half a century [1]. The symptoms of CCS are characterized by the presence of ectodermal abnormalities, including alopecia, onchodystrophy and cutaneous hyperpigmentation, and they also associated with prominent clinical features, including diarrhea, weight loss, hypogeusia, anorexia and intestinal malabsorption [1,3]. In the present case as well as other reported cases, endoscopic characteristics of CCS were impressive (Figures 1, 2 and 3) and histological examination of polyps showed diversity in appearance: inflammatory, hyperplastic and adenomatous types. Although CCS is considered a benign condition, the occurrence of cancer in the stomach and colorectum of CCS patients is about 13% (50/387), and there is a possibility of serrated adenoma-carcinoma sequence in colorectal cancer [4].

Many complications such as fatal gastrointestinal bleeding, intussusception, electrolyte abnormalities, protein-losing enteropathy, and severe acute pancreatitis may occur with CCS, possibly contributing to poor outcomes [3,5-8]. Interestingly, the present case was complicated by rib fractures, which, to our knowledge, has not been reported in the published literature. Since our patient had no medical history of trauma, persistent cough, chest pain and congenital disease, and our examinations did not establish the definite etiology of the rib fractures. In addition, low dosage prednisone for 24 days, in our opinion, could not have resulted in the multiple rib fractures. Therefore, we presume that persistent hypocalcaemia and malnutrition contribute to this situation, or at least aggravate this rare complication.

Although it is believed that no specific treatment has consistently resulted in definite improvement, current literature favors a combination therapy, which could induce remission as seen in our patient, based on nutritional support and corticosteroids [9]. Since prolonged corticosteroid therapy will result in an increased risk of osteoporotic fracture, CCS patients who accept corticosteroid treatment should be monitored for the potential harmful complications such as rib fracture.

Although the overall mortality has been reported as high as 55% according to an early study [10], the prognosis is now thought to be better than earlier case reports, with improvement in medical treatment and increased understanding of the syndrome.

## Conclusions

This is the first case of a CCS patient with multiple rib fractures. Although the association between CCS and multiple rib fractures in this case remains uncertain, we presume that persistent hypocalcaemia and malnutrition contribute to this situation, or at least aggravate this rare complication. Besides, since prolonged corticosteroid therapy will result in an increased risk of osteoporotic fracture, CCS patients who accept corticosteroid for treatment could be potential victims of rib fracture.

## Consent

Written informed consent was obtained from the patient for publication of this case report and any accompanying images. A copy of the written consent is available for review by the Editor-in-Chief of this journal.

## Abbreviations

CCS: Cronkhite-Canada syndrome.

## Competing interests

The authors declare that they have no competing interests.

## Authors' contributions

YB reviewed the literature and drafted the manuscript; JX, LH, SY and ZR created figures, collected clinical data and analyzed data; LJ, JX, WH and LH were involved in the care of the patient, WF performed the endoscopy; SY, ZX and WF revised the manuscript. All authors read and approved the final manuscript.

## Pre-publication history

The pre-publication history for this paper can be accessed here:

http://www.biomedcentral.com/1471-230X/10/121/prepub
